# Heritability of Radiation Response in Lung Cancer Families

**DOI:** 10.3390/genes3020248

**Published:** 2012-03-29

**Authors:** Albert Rosenberger, Ute Rössler, Sabine Hornhardt, Wiebke Sauter, Heike Bickeböller, H.-Erich Wichmann, Maria Gomolka

**Affiliations:** 1 Department of Genetic Epidemiology, University Medical Center, Georg-August-University Göttingen, Humboldtallee 32, 37073 Göttingen, Germany; E-Mail: hbickeb@gwdg.de; 2 Department of Radiation Protections and Health, Federal Office for Radiation Protection, Ingolstaedter Landstr.1, 85764 Oberschleissheim, Germany; E-Mails: uroessler@bfs.de (U.R.); shornhardt@bfs.de (S.H.); wiebke.sauter@gmx.de (W.S.); mgomolka@bfs.de (M.G.); 3 Institute of Epidemiology, Helmholtz Center Munich, 85764 Munich, Germany; E-Mail: wichmann@helmholtz-muenchen.de; 4 Department of Medical Informatics, Biometry and Epidemiology, Ludwig-Maximilians-University Munich, 80539 Munich, Germany

**Keywords:** COMET Assay, DNA damage, familial aggregation, lung cancer

## Abstract

Radiation sensitivity is assumed to be a cancer susceptibility factor due to impaired DNA damage signalling and repair. Relevant genetic factors may also determine the observed familial aggregation of early onset lung cancer. We investigated the heritability of radiation sensitivity in families of 177 Caucasian cases of early onset lung cancer. In total 798 individuals were characterized for their radiation-induced DNA damage response. DNA damage analysis was performed by alkaline comet assay before and after *in vitro* irradiation of isolated lymphocytes. The cells were exposed to a dose of 4 Gy and allowed to repair induced DNA-damage up to 60 minutes. The primary outcome parameter Olive Tail Moment was the basis for heritability estimates. Heritability was highest for basal damage (without irradiation) 70% (95%-CI: 51%–88%) and initial damage (directly after irradiation) 65% (95%-CI: 47%–83%) and decreased to 20%–48% for the residual damage after different repair times. Hence our study supports the hypothesis that genomic instability represented by the basal DNA damage as well as radiation induced and repaired damage is highly heritable. Genes influencing genome instability and DNA repair are therefore of major interest for the etiology of lung cancer in the young. The comet assay represents a proper tool to investigate heritability of the radiation sensitive phenotype. Our results are in good agreement with other mutagen sensitivity assays.

## 1. Introduction

Lung cancer (LC) is the most common cause of cancer death worldwide with a 5-year survival probability of only 10% [1]. Although tobacco smoking remains the predominant cause of LC, even in never smokers LC is an important public health issue. It is estimated that 10% to 29% of LC cases are attributable to factors other than smoking, representing between 16,000 and 24,000 LC deaths annually in the US alone [2,3,4]. To date research studies were able to identify only a few genomic loci which are significantly associated with LC risk and confirmed in several investigations [5,6]. However a pooled analysis of 14 studies revealed weak associations of borderline significance of some genomic variants related to DNA repair and cell cycle [7]. The fact that a 3-fold risk for LC was found for first degree relatives in LC patients younger than 46 years of age supports the hypothesis of a genetic component in the etiology of early onset lung cancer [8]. Even a 7-fold risk within non-smokers younger than 60 was reported [9]. A segregation analysis within 337 families showed that 70% of the early onset LC risk (patients younger than 50 years) can be attributed to rare autosomal genes [10,11]. Another indication for a genetic contribution to lung cancer in the young is given by a larger increase of risk in monozygotic twins compared to dizygotic twins. This was more evident in female than in male twins [12]. No risk differences could be detected in a cohort of twins older than 50 [13] indicating the strong effect of age for the genetic component of this disease.

Cancer risk is also often associated with radiation sensitivity. Numerous publications demonstrated clearly that DNA repair is impaired in cancer patients [14] compared to the normal population. Thus impaired DNA repair results in a higher susceptibility to cancer [15]. This has been detected in individuals with deficiencies in one or more genes of the DNA repair systems or cell cycle checkpoints resulting in a greater sensitivity to mutagen challenge e.g., radiation. Examples are gene defects in ATM [16], NBS1 [17], BRCA1 and BRCA2 [18] or LIG4 [19]. Individuals harbouring mutations in these genes show a deficiency in cellular survival after radiation challenge, defective DNA repair and/or increased chromosomal damage. Mutagen sensitivity represents therefore a cancer susceptibility factor that is highly heritable [20]. 

The existence of considerable inter-individual variation in DNA damage induction and DNA repair capacity is widely accepted. However, the estimated heritability of several cellular effects based on DNA damage, like chromosomal or chromatid damage [20,21,22], micronucleus induction [23], apoptosis induction [24] and repair of DNA breaks [25], range from 60% to 80%. This indicates that genetic factors contribute substantially to the observed phenotypic variation. The aim of our study was to estimate for the first time the heritability of the radiation sensitive phenotype in families of early onset lung cancer patients.

Our molecular epidemiological study was conducted on blood samples of a well described sample population of 175 young cancer patients (**LU**ng **C**ancer in the **Y**oung **LUCY**) and 623 relatives (mother, father, siblings and children) and spouses. We assumed that genetic factors influence radiation induced DNA damage and repair capacity to a high degree. High throughput COMET assays including an automated cell analysis step were performed to investigate basal DNA damage as well as initial DNA damage and repair in such a large sample [26]. The COMET assay detects gamma-radiation induced single- (SSB) and double strand breaks (DSB) induced by ionizing radiation on a single cell basis [27,28].

## 2. Results and Discussion

The family component of the study comprised 177 index-persons (cases of early onset lung cancer) with a mean age of 44.2 years (all being diagnosed before the age of 50), whereof 99 (57%) were males. In addition, information of 180 parents (in 123 families), 276 siblings (in 152 families), 63 partners and 104 adult offspring (in 80 families) was available (see Supplementary Table 1). All relationships were in concordance with Mendalien segregation proofed by 68 SNPs (data not shown). The proportion of non-smokers was lowest in index-persons (11%), followed by partners and siblings (27%) and highest within parents and children (47%). In the German population the proportion of non-smokers is about 75% [29]. Thus smokers are more frequent in our study families. We used information of 133 families comprising two generations and further 35 families comprising three generations (Table 1). 

**Table 1:** Types of families in the study population.

*Generations*	*Composition of family*					*Total*	
	*Parents*	*LC case*	*Siblings*	*Partners*	*Offspring*		
1 (unsuitable for the estimation of heritability)	--	1	--	--	--	1	9
	--	1	--	≥1	--	1	
	--	1	≥1	≥1	--	7	
2	--	--	≥1	≥1	≥1	1	133
	--	1	--	--	≥1	4	
	--	1	--	≥1	≥1	9	
	--	1	≥1	≥1	≥1	31	
	1	--	--	≥1	--	1	
	1	1	--	--	--	6	
	1	1	--	≥1	--	35	
	1	1	≥1	≥1	--	3	
	2	1	--	--	--	18	
	2	1	--	≥1	--	24	
	2	1	≥1	--	--	1	
3	1	1	--	--	≥1	1	35
	1	1	--	≥1	≥1	10	
	1	1	≥1	--	≥1	1	
	1	1	≥1	≥1	≥1	9	
	2	1	--	--	≥1	1	
	2	1	--	≥1	≥1	3	
	2	1	≥1	--	≥1	3	
	2	1	≥1	≥1	≥1	7	
*Total*							*177*

### 2.1. DNA Damage and DNA Repair

We found a significant difference between healthy controls and early onset lung cancer patients with respect to DNA repair capacity in a separate and independent case control study applying an identical comet assay protocol [30]. These data support the hypothesis that impaired repair capacity is also detectable in our patient cohort, may be due to genetic factors.

**Figure 1:**
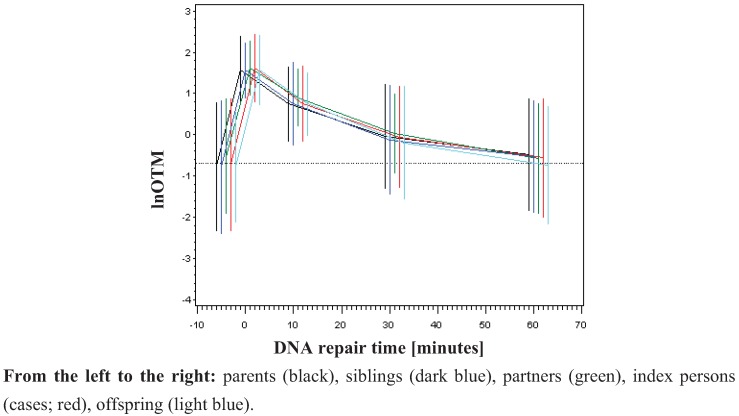
Time kinetic of DNA damage and repair by groups of relatives (mean and ±std. dev.)

To analyse DNA damage in the family design, the primary outcome parameter OTM was used for the analysis of basal damage, initial DNA damage and residual DNA damage after repair. The logarithm of the Olive Tail Moment (OTM) was taken to transform the data set into a normal distribution function [26,31,32,33], which was shown to be highly sensitive for the evaluation of DNA damage [26]. No difference between parents, index person, siblings, offspring or partners was detected (Figure 1). The measured basal damage (not radiation exposed) was on average −0.74 (std. dev. 0.77). Directly after irradiation lnOTM was on average 1.59 (std. dev. 0.36). DNA repair was almost completed after 60 minutes (Figure 1) as demonstrated by an average lnOTM declining to −0.56 (std. dev. 0.70). If genetic factors do influence DNA damage one may expect to observe DNA damage declining from the patient group, to the siblings, parents and children and DNA damage should be lowest in spouses due to the degree of relationship. Here, we found no differences among the investigated groups neither for the basal damage nor for the induced radiation DNA damage or for residual DNA damage (Figure 1 and Supplementary Table 3). Probably the supposed effect is too small to be resolved in this family study design. In contrast, Rajeswari *et al*. [25] found significant differences in the DNA damage response detected by the alkaline COMET assay among breast cancer patients, first degree relatives and controls at baseline levels as well as for induced damage and repair efficiency when human lymphocytes were treated with the mutagen N-methyl N-nitro N-nitrosoguanidine. 

Legend to Figure 1: DNA damage is expressed in [lnOTM]. On X-axes the different time points are given. 0 represents the initial damage after irradiation. Residual damage was detected after 10, 30 and 60 minutes after irradiation. The non-irradiated controls are represented in the time point before irradiation (0). 

### 2.2. Impact of Age, Sex and Smoking

**Figure 2:**
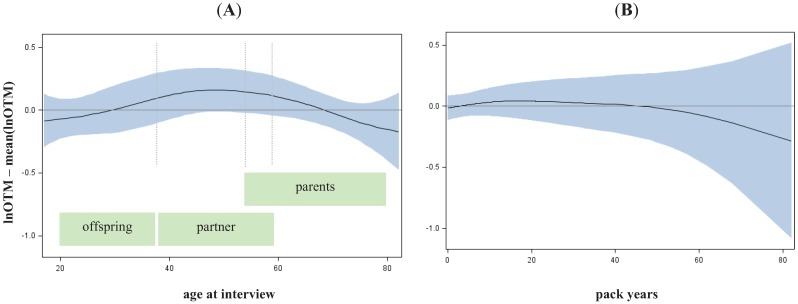
Alteration of basal damage along age (**A**) and pack years (**B**). Spline with df = 2 and 95%–confidence interval adjusted for age, gender and smoking.

To estimate if heritability was modified by other factors, we investigated the impact of age, gender and smoking on the observed DNA damage as detected by the damage parameter lnOTM. Only age was found to have a significant but weak effect on basal damage (p = 0.0439), and DNA repair after 10 minutes (p = 0.0286) and 60 minutes (p = 0.0179) while gender and smoking remained not significant. No alteration at all could be observed regarding the initial damage (see Supplementary Table 4 and Figure 2). However, the age dependent effect of the basal damage might be less a biological phenomenon, rather than an effect of residual confounding. Interestingly the group of middle aged individuals (40–60 years), which contained the majority of partners of LC cases, showed a higher basal damage compared to groups of older (mainly containing parents) and younger (mainly containing offspring) individuals. However, the age-dependent increase of basal damage could be a consequence of residual confounding, e.g., by passive smoking or sub-adequate quantification of long life smoking behaviour. More siblings and partners (enriched in middle ages) were smokers (proportion of never smokers is 27% and 25% respectively), than children (enriched in lower ages, proportion of never smokers 43%) and parents (enriched in higher ages, proportion of never smokers 47%). Therefore, we assumed that the estimation of heritability will not be noticeable biased by age, sex or smoking.

Legend to Figure 2: Lung cancer patients were disregarded to avoid bias due to disease status.

### 2.3. Heritability

Heritability was estimated for the basal damage, the initial radiation-induced damage, and residual damage of all available two- and three-generation families. We used parent-offspring regression to estimate the nominal heritability (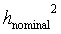) [34,34,35,36]. Depending on the availability of parental outcome measures we performed mid-parent-offspring regression or one parent-offspring regression. Because the COMET assay is an experiment affected by numerous noisy or confounding factors [26], we assessed the effect of the experimental design onto the estimation of heritability. Hereby we introduced a falsity-correction for 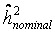
considering pseudo-families generated from independent controls of a control cohort (KORA). All controls within a single trial were randomly grouped in pseudo-families, where the status as pseudo-parent or pseudo-offspring was assigned by chance. Heritability was estimated as described above in each of 3000 repetitions. The mean estimate was considered as falsity 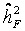
induced by study and assay design. For internal validation we investigated spouse-correlation in terms of spearman’s rank correlation of lnOTM between partners and parents, siblings and the index person respectively, separately for basal damage, induced damage and residual damage.

We found a mean heritability in pseudo-families of about 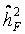
= 20%, which was used as falsity-correction factor. Hence, an adjustment of the primary outcome parameter by repeated measurement of a single human blood donor (an identical human reference sample used in every experimental set) is insufficient in clearing interfering design or assay specific factors [26]. However, heritability in pseudo-families ranges from 4% to 28% (Table 2), indicating the methodological difficulties for an appropriate correction. This range might be due to experimental variability at short time points of DNA repair and the large proportion of white noise in the radiation induced damage [26].

**Table 2:** Heritability of DNA damage and repair.

	Falsity-correction 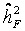	Nominal heritability 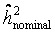	Corrected heritability 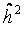
	*pseudo-families*	*LC-families*	*LC-families*
Basal damage	23%	*77% (63%–91% )*	70% (52%–88%)
Initial damage	28%	*75%(62%–88% )*	65% (47%–83%)
Repair 10 min	22%	*56% (45%–67% )*	44% (29%–58%)
Repair 30 min	18%	*34% (24%–43% )*	20% (07%–30%)
Repair 60 min	4%	*50% (36%–65% )*	48% (33%–64%)

The corrected heritability of the basal damage and DNA damage directly after irradiation were about 70% (95%-CI: 51%–88%) and 65% (95%-CI: 47%–83%), respectively. However, during the repair period (10–60 min after irradiation) we observed a nominal decreased heritability between 20% and 48%. We observed no significant spouse correlation, except to offspring, as expected (see Supplementary Table 5). 

These estimates of heritability are in accordance with observations of several other studies. Already 1963 Roderick *et al*. estimated a heritability of 30% to 50% for survival after radiation exposure in mice. Roberts *et al*. [37] analysed 95 family members of radiation sensitive breast cancer patients and could demonstrate a clear evidence of heritability of 82% in the chromosomal G2 radiation sensitivity assay, assuming a single major gene effect. In a classical twin study design, the genetic and environmental variance components were estimated on the basis of the mutagen sensitivity assay [20], which is able to detect chromatid breaks after mutagen challenge. Among various mutagens, a genetic heritability of 62.5% after gamma irradiation was estimated. This heritability was highest compared to the investigated chemical mutagens in the study. The heritability of mutagen sensitivity is further supported by several other twin and sib pair studies considering, e.g., micronuclei frequency [23] as an additional endpoint. The heritability for baseline micronuclei frequency was found to be between 68% and 72% that of radiation induced micronuclei frequency between 57% and 68%. This is in agreement with our COMET assay results. Familial correlations of radiation induced apoptosis among first-degree relatives (father-mother-offspring trios) were observed for different lymphocyte populations (B- and T-cells) [24]. A heritability of 43% for the radiation sensitive phenotype of T4 effector memory cells was estimated. The large heritability of the basal damage indicates that DNA stability has a genetic component, irrespectively of experimental irradiation. This finding is especially supported by twin studies which rules out shared environmental effects [23,25]. 

The observed decrease of heritability of the damage after repair may be interpreted by a decreased genetic component as supported also by others [23], but it could also be due to an enhanced inherent experimental variation in determining DNA repair which results in decreased heritability estimates. We conclude that not only radiation induced damage is determined by genetic factors, already the level of basal DNA damage has a strong genetic component. This parameter is directly related to genome stability. Genomic instability on the other hand was shown to be a major factor in the chromosomal radiosensitivity [14]. Our findings support the epidemiological observations that a genetic component is involved in the etiology of early onset of lung cancer. For future studies it is important to identify genes contributing to genomic instability which are not necessarily involved in DNA repair. 

## 3. Experimental Section

### 3.1. Study Groups

Samples of fresh peripheral blood (EDTA) were taken from participants of the LUCY Study (Lung Cancer in the Young, [38], 355 LC patients–diagnosed prior to the age of 51 years–623 relatives of 175 families, and 170 population controls free of cancer (KORA Study: *KO*operationsstudie im *R*aum *A*ugsburg [39,40]. Blood samples of LC were taken at the time of primary diagnosis before any treatment. Results of the so called family part of the study (LC patients as index probands and their relatives) are reported here.

Independent LC cases and controls (comprised in the case-control part) were used for bias correction. Further details are described elsewhere [38,41]. In addition to these study groups, isolated peripheral blood lymphocytes out of EDTA blood samples taken from a single healthy male collected from three donations over the time period were used in all assays as human reference (HR) [26]. Isolated cell aliquots were stored in liquid nitrogen and used over the whole investigation period. 

### 3.2. Isolation of Lymphocytes, Irradiation and DNA Repair

Lymphocytes were isolated from blood samples by Ficoll separation (Leucosep) and cryopreserved. For the *COMET assay*, lymphocytes were thawed quickly and incubated for 24 hours. Aliquots were exposed *in vitro* to 4 Gy of gamma radiation on ice. Irradiated aliquots were set on ice prior to embedding in agarose to avoid repair of DNA damage. DNA repair was assessed by incubation of the cell samples at 37 °C after irradiation. Aliquots were taken after 10, 30 or 60 minutes after incubation and set on ice. In parallel, sham-irradiated controls were also collected after the same time intervals to measure basal damage for control. A detailed protocol description is given elsewhere [26].

### 3.3. Exposure Level and Batch Processing, COMET Assay, Image Acquisition and COMET Analysis

Irradiation and DNA repair are jointly denoted as exposure levels: *basal damage* (0 Gy)–*initial irradiation-damage* (4 Gy/0 min)–*repair 10 min* (4 Gy/10 min)–*repair 30 min* (4 Gy/30 min) and *repair 60 min* (4 Gy/60 min). Batches of aliquots of patients and their relatives were preceded together, denoted as one trial. Each sample was studied under each of the exposure levels at least once. In total, 173 trials were completed, containing 10,016 single experiments (see Supplementary Table 2) with data of about 1,750,000 single cells as base outcome measures.

The COMET assay and the equipment are described in detail in [26].

### 3.4. Measures for DNA Damage

The logarithm of the Olive Tail Moment (lnOTM) was investigated as the base outcome metric per cell nucleus. The basal damage (B) corresponds to the primary metric for no irradiation (0 Gy). The initial radiation-induced damage (I) corresponds to the primary metric directly after irradiation (4 Gy after 0 minutes’ repair time) minus the basal damage: The DNA repair capacity (DRC) (secondary outcome measure) was determined for 10, 30, and 60 minutes after irradiation. DRC is defined as the non-repaired proportion of the initial radiation-induced damage I at a specific time point, corrected by the basal damage B. Additional to blood samples of cases, controls and relatives, samples of a single individual (denoted as human reference HR) were processed and measured in the same way throughout the whole investigation. We assumed, that the empirical variance of outcome measures of HR reflects the blurring effects of design-inherent endogenous factors (white noise). Comparing measure variability of cases, controls and relatives with that of HR, we recognised that a proportion of 7% to 95% can be attributed to assay variation (white noise). This differed between exposure level and study group.

After thoroughly univariate and multivariate inspection of design factors we finally used generalized additive models, which provide a high level of flexibility in modelling, to reduce white noise. Spline estimators for duration of measurement, date of survey, number of non-selected cells and position, such as the temporal progression of the long lasting investigation were performed were finally use gain adjusted measurements. More details of study groups, material preparation, performing and analysing the assay and deriving outcome measures are given elsewhere [26].

### 3.5. Impact of Age, Sex and Smoking

A modification of the primary outcome by age, sex and smoking was investigated by fitting semi-parametric linear models. For age and pack years a spline function with two degrees of freedom was fitted, for sex a single parameter. To avoid bias correlated to the status as lung cancer patients, indexpersons where excluded from the model fit.

### 3.6. Estimation of Heritability

Heritability was estimated for the basal damage (B), the initial radiation-induced damage (I), and all three DNA repair capacities (DRC) of all available two- and three-generation families. We used parent-offspring regression to estimate the nominal heritability 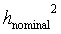 [34,36,42]. Depending on the availability of parental outcome measures we performed mid-parent-offspring regression (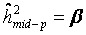
if measures of both parents were available) or one parent-offspring regression (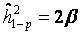
if measures of only one parents were available), where 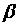
is the regression coefficient of a linear regression, adjusted for the offspring’s mean age and mean gender and the value of a human control to adjust for inter-assay variability. Both heritability estimates were than pooled by 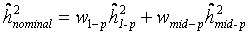 (weighted mean) with weights equal to the inverse of the estimated sample variance
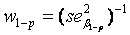
and 
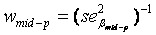
. The standard error of 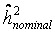
was calculated as 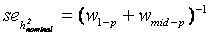 [43].

Because the COMET assay is an experiment affected by numerous noisy or confounding factors [26], we estimated a falsity-correction for 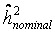
considering independent controls. All controls within a single trial were randomly grouped in pseudo-families, where the status as pseudo-parent or pseudo-offspring was assigned by chance. Heritability was estimated as described above in each of 3000 repetitions. The mean estimate was considered as falsity 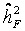
induced by study and assay design. Depending on the range of values for heritability spans from 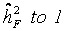 (instead of 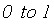) the final estimate is given by 
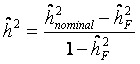
.

For internal validation we investigated spouse-correlation in terms of spearman’s rank correlation of lnOTM between partners and parents, siblings and the index person respectively, separately for B, I and DRC.

## 4. Conclusions

Our study supports the hypothesis that radiation sensitivity is highly heritable as shown by the Comet assay. The estimates of heritability are in good agreement with findings based on other mutagen sensitivity assays, like micronuclei induction, G2-assay and apoptosis induction. For the etiology of lung cancer in the young our data support that not only repair mechanisms after mutagen exposure like ionizing radiation are of major interest but also genetic factors determining genome stability.

## Supplementary Files

Supplementary File 1 PDF-Document (PDF, 158 KB)
