# Supplementary material for: Heritability of Radiation Response in Lung Cancer Families
Source: Genes (Basel). 2012 Mar 29;3(2):248–60. doi: 10.3390/genes3020248 (PMC3899950; doi:10.3390/genes3020248)
Supplement: Supplementary File 1 — PDF-Document (PDF, 158 KB) [file genes-03-00248-s001.pdf]

Genes **2011**, 3, 1-3

OPEN ACCESS

genes

ISSN 2073-4425

www.mdpi.com/journal/genes

Article

Heritability of Radiation Induced DNA Damage Response in Lung Cancer Families

Albert Rosenberger [1,*], Ute Rössler [2], Sabine Hornhardt [2], Wiebke Sauter [2], Heike Bickeböller [1], H.-Erich Wichmann [3,4] and Maria Gomolka [2]

Supplementary Material

Table 1. Characteristics of study participants.

		Parents (n = 180)			*Siblings (n = 276)*			*Partners (n = 63)*			*Probands (n = 177)*			*Offspring (n = 104)*		
		N			***N***			***N***			***N***			***N***		
Sex	*unknown*	9	5%		8	3%					4	1%		3	3%	
	male	65	36%		121	44%		22	35%		99	57%		51	49%	
	female	106	59%		147	53%		41	65%		74	42%		50	48%	
		N	***mean***	***s***	***N***	***mean***	***s***	***N***	***mean***	***s***	***N***	***mean***	***s***	***N***	***mean***	***s***
Age		*171*	*69.3*	*6.3*	*270*	*44.6*	*7.7*	*63*	*46.3*	*5.0*	*173*	*44.2*	*4.7*	*103*	*21.8*	*3.9*
Smoking status	*unknown*	*11*	*6%*		*12*	*4%*		*1*	*2%*		*4*	*1%*		*2*	*2%*	
	Never smoker	84	47%		74	27%		16	25%		20	11%		45	43%	
	Occasional smoker [$]	1	1%		3	1%		1	2%		1	1%		2	2%	
	Former smoker	51	28%		68	25%		18	29%		26	15%		2	2%	
	Current smoker	33	18%		119	43%		27	43%		126	72%		53	51%	
		N	***mean***	***s***	***N***	***mean***	***s***	***N***	***mean***	***s***	***N***	***mean***	***s***	***N***	***mean***	***s***
Age at first Cigarette	*total*	*85*	*21.9*	*9.1*	*190*	*17.5*	*4.0*	*46*	*17.9*	*4.2*	*153*	*16.8*	*3.6*	*57*	*15.7*	*2.4*
	male	48	19.6	4.9	89	17.2	3.7	15	18.0	2.3	91	16.9	3.9	32	15.5	2.4
	female	37	24.9	12.0	99	17.8	4.2	31	17.9	4.9	62	16.5	3.1	24	16.1	2.4
Pack years	*total*	*168*	*10.6*	*16.0*	*253*	*12.8*	*16.5*	*58*	*10.9*	*14.9*	*166*	*21.0*	*15.9*	*98*	*2.6*	*4.3*
	male	64	16.4	18.5	112	16.0	20.2	21	16.6	21.3	93	24.0	15.9	49	3.1	4.6
	female	104	7.0	13.0	139	10.2	12.1	37	7.7	8.3	73	17.1	15.1	47	2.1	3.9

[$] Occasional smoker are smokers with less than 1 pack year, having smokes for longer than 1 year. s Standard deviation.

Table 2. Number of individuals, trials and experiments.

	Experiments	*Trials*	*Individuals*
LC patient	3015	156	355
Human reference [a]	1423	164	1
Controls	1601	66	170
Relatives	3977	77	610
Total	10016	173	1136

[a] in 9 trials no valid measures of the Human reference sample could be achieved.

Table 3. Radiation sensitivity (lnOTM and DNA repair capacity)

	Parents				*Siblings*				*Offspring*			
	n	*mean*	*s*	*median*	*n*	*mean*	*s*	*median*	*n*	*mean*	*s*	*median*
lnOTM												
Basal damage	173	−0.77	0.78	−0.9	271	−0.72	0.80	−0.9	100	−0.79	0.66	−0.9
Initial damage	178	1.58	0.40	1.6	275	1.61	0.41	1.6	103	1.57	0.42	1.6
Repair 10 min	176	0.75	0.45	0.7	274	0.75	0.46	0.7	104	0.74	0.38	0.7
Repair 30 min	174	−0.04	0.63	−0.0	275	−0.06	0.61	−0.0	104	−0.20	0.68	−0.1
Repair 60 min	175	−0.48	0.68	−0.5	274	−0.56	0.72	−0.6	101	−0.74	0.71	−0.7
DRC												
Repair 10 min	164	0.44	0.49	0.4	270	0.45	0.76	0.4	99	0.37	0.19	0.4
Repair 30 min	165	0.81	0.61	0.7	271	0.89	1.96	0.7	99	0.76	0.32	0.7
Repair 60 min	165	1.01	0.55	0.9	270	1.14	2.21	0.9	98	0.99	0.40	0.9
	Partner				***Probands***				***total***			
	n	*mean*	*s*	*median*	*n*	*mean*	*s*	*median*	*n*	*mean*	*s*	*median*
lnOTM												
Basal damage	61	−0.52	0.69	−0.6	160	−0.79	0.81	−1.0	765	−0.74	0.77	−0.9
Initial damage	63	1.62	0.33	1.6	172	1.56	0.33	1.5	791	1.59	0.39	1.6
Repair 10 min	63	0.90	0.35	0.9	174	0.76	0.50	0.7	791	0.76	0.45	0.7
Repair 30 min	63	0.03	0.47	−0.0	169	−0.12	0.66	−0.1	785	−0.08	0.63	−0.1
Repair 60 min	63	−0.58	0.67	−0.5	169	−0.52	0.68	−0.5	782	−0.56	0.70	−0.6
DRC												
Repair 10 min	61	0.35	0.36	0.4	155	0.38	0.29	0.3	749	0.41	0.54	0.4
Repair 30 min	61	0.67	1.32	0.7	155	0.81	0.65	0.7	751	0.82	1.30	0.7
Repair 60 min	60	0.99	0.97	1.0	152	1.00	0.56	0.9	745	1.05	1.42	0.9

s Standard deviation, DRC.DNA- repair capacity.

Table 4. Impact of age, sex and smoking according a generalize additive model.

	p-values		
	age (Spline)	*Pack years (Spline)*	*sex*
Basal damage	* 0.0439	0.1983	0.4026
Initial damage	0.2241	0.3073	0.6550
Repair 10 min	* 0.0286	0.2445	0.5157
Repair 30 min	0.1227	0.1612	0.4401
Repair 60 min	* 0.0179	0.0887	0.5683

Table 5. Spouse correlation.

	Relationship to index person	***Rank correlation (95%-CI)***		***p-value***	***N***
Basal damage	*offspring*	0.39	(0.13–0.60)	0.0041	50
Basal damage	*Parents*	0.01	(−0.43–0.45)	0.9506	20
Basal damage	*siblings*	0.13	(−0.16–0.40)	0.3868	48
Basal damage	*Index-person*	0.09	(−0.19–0.36)	0.5246	51
Initial damage	*offspring*	0.57	(0.35–0.73)	<0.0001	52
Initial damage	*Parents*	0.21	(−0.22–0.56)	0.3276	24
Initial damage	*siblings*	0.22	(−0.04–0.45)	0.0986	57
Initial damage	*Index-person*	0.23	(−0.02–0.46)	0.0661	62
Repair 10 min	*offspring*	0.45	(0.20–0.65)	0.0008	50
Repair 10 min	*Parents*	0.30	(−0.21–0.68)	0.2323	17
Repair 10 min	*siblings*	0.22	(−0.07–0.48)	0.1280	47
Repair 10 min	*Index-person*	0.23	(−0.06–0.47)	0.1112	50
Repair 30 min	*offspring*	0.35	(0.08–0.58)	0.0106	50
Repair 30 min	*Parents*	0.48	(0.02–0.77)	0.0368	18
Repair 30 min	*siblings*	0.20	(−0.09–0.47)	0.1723	46
Repair 30 min	*Index-person*	0.19	(−0.09–0.45)	0.1799	49
Repair 60 min	*offspring*	0.22	(−0.06–0.47)	0.1233	49
Repair 60 min	*Parents*	0.41	(−0.08–0.73)	0.0864	18
Repair 60 min	*siblings*	0.22	(−0.08–0.48)	0.1492	45
Repair 60 min	*Index-person*	0.13	(−0.16–0.41)	0.3674	47
